# Detection of Selected Extended‐Spectrum β‐Lactamase (ESBL) Genes in *Escherichia coli* Isolated From Dogs at Juanda Quarantine Station, Surabaya, Indonesia

**DOI:** 10.1155/vmi/3595305

**Published:** 2026-07-28

**Authors:** Benny Aprisa Surya Perdana, Mustofa Helmi Effendi, Wiwiek Tyasningsih, Wiwik Misaco Yuniarti, Boedi Setiawan, Dadik Rahardjo, Fidi Nur Aini Eka Puji Dameanti, John Yew Huat Tang

**Affiliations:** ^1^ Veterinary Medicine and Public Health, Faculty of Veterinary Medicine, Universitas Airlangga, Jl. Dr. Ir. H. Soekarno Kampus C Mulyorejo, Surabaya 60115, East Java, Indonesia, unair.ac.id; ^2^ Department of Veterinary Public Health, Faculty of Veterinary Medicine, Universitas Airlangga, Jl. Dr. Ir. H. Soekarno Kampus C Mulyorejo, Surabaya 60115, East Java, Indonesia, unair.ac.id; ^3^ Research Group on Antimicrobial Resistance, Faculty of Veterinary Medicine, Universitas Airlangga, Jl. Dr. Ir. H. Soekarno Kampus C Mulyorejo, Surabaya 60115, East Java, Indonesia, unair.ac.id; ^4^ School of Food Industry, Faculty of Bioresources, and Food Industry, Besut Campus, Universiti Sultan Zainal Abidin, Jl. Tembila, Besut 22200, Terengganu, Malaysia, unisza.edu.my; ^5^ Department of Veterinary Microbiology, Faculty of Veterinary Medicine, Universitas Airlangga, Jl. Dr. Ir. H. Soekarno Kampus C Mulyorejo, Surabaya 60115, East Java, Indonesia, unair.ac.id; ^6^ Department of Veterinary Clinic, Faculty of Veterinary Medicine, Universitas Airlangga, Jl. Dr. Ir. H. Soekarno Kampus C Mulyorejo, Surabaya 60115, East Java, Indonesia, unair.ac.id; ^7^ Laboratory of Microbiology and Immunology Veterinary, Faculty of Veterinary Medicine, Universitas Brawijaya, Jl. Puncak Dieng Kalisongo, Malang Regency 65151, East Java, Indonesia, ub.ac.id

**Keywords:** dog, ESBL, gene detection, Indonesia, quarantine

## Abstract

Currently, global health is threatened by the issue of antibiotic resistance, with companion animals potentially serving as reservoirs of extended‐spectrum β‐lactamase (ESBL) genes. This study aimed to detect selected ESBL genes in *Escherichia coli* isolated from dogs undergoing interregional transport procedures at Juanda Quarantine Station, Surabaya, Indonesia. This study was conducted from March to May 2025 at Juanda Quarantine Station, Surabaya, Indonesia. Rectal swab samples collected from 113 dogs were preenriched in Buffered Peptone Water (BPW), followed by isolation and identification of Escherichia coli. Selected ESBL genes (*blaCTX-M*, *blaTEM*, and *blaSHV*) were detected by polymerase chain reaction (PCR). The identification results showed that 75.22% (85/113) were positive for *Escherichia coli*. Among these isolates, 3 (3.53%) carried ESBL genes, with 2/3 (66.67%) harboring a combination of *blaCTX-M* and *blaTEM* and 1/3 (33.33%) carrying *blaCTX-M* alone. No *blaSHV* gene was detected. Although the prevalence was relatively low, these findings indicate that dogs may serve as carriers of ESBL‐producing *Escherichia coli* and may contribute to the dissemination of antimicrobial resistance. These results underscore the importance of prudent antibiotic use, strengthened antimicrobial stewardship, and a One Health approach to mitigate the spread of antimicrobial resistance among humans, animals, and the environment.

## 1. Introduction

Extended‐spectrum β‐lactamases (ESBLs) are enzymes commonly produced by Gram‐negative bacteria, particularly members of the family Enterobacteriaceae [1]. This enzyme acts by inactivating beta‐lactam antibiotics, such as penicillins, monobactams, and first‐, second‐, and third‐generation cephalosporins, through hydrolysis of the β‐lactam ring [2]. The administration of β‐lactam antibiotics for bacterial infection control includes penicillins, cephalosporins, monobactams, and carbapenems [3]. Plasmids harboring ESBL‐encoding genes often contain additional resistance determinants against fluoroquinolones, aminoglycosides, and sulfonamides; thus, ESBL‐producing strains are commonly resistant to multiple classes of antibiotics. This greatly limits therapeutic options for treating bacterial infections and contributes to increased mortality, morbidity, and hospital costs [4]. Among Gram‐negative bacteria present in environmental samples, *Escherichia coli* is the most frequently encountered species, with *blaCTX-M* and *blaTEM* genes being the predominant ESBL determinants, highlighting its role as a key reservoir for antimicrobial resistance [5, 6]. While most strains of *Escherichia coli* are harmless, certain pathogenic strains can cause disease depending on the virulence factors present [7].

The spread of ESBL genes is facilitated by mobile genetic elements such as integrons, plasmids, and transposons, which enable both vertical and horizontal transmission of antimicrobial resistance determinants [8]. The use of antimicrobial agents in animals, including companion animals, often parallels their use in humans, increasing the risk of selection and dissemination of resistant bacteria [9]. In companion animals, particularly dogs, pathogenic and commensal bacteria may develop antimicrobial resistance following antimicrobial exposure, or resistant bacteria may be acquired from humans or the environment [10]. Environments with high microbial density may serve as reservoirs or facilitators of horizontal gene transfer, thereby enabling the dissemination of resistance genes among bacterial populations [11]. Because dogs live in close contact with humans, they may also contribute to the transmission of resistant bacteria and resistance genes to their owners [12]. In addition, the frequent movement of dogs across regions may facilitate the dissemination of resistant bacteria to new geographic areas, emphasizing the relevance of antimicrobial resistance surveillance in companion animals within a One Health framework.

Previous studies have reported the occurrence of ESBL‐producing *Escherichia coli* in dogs in different settings. A global review showed that ESBL‐producing *Escherichia coli* is widespread among companion animals, with an estimated prevalence of 6.87% in dogs globally and 6.21% in dogs in Europe. The review also identified CTX‐M‐type ESBLs and ST131 among the most widely distributed ESBL genes and sequence types worldwide [13]. In Indonesia, rectal swabs collected from dogs at veterinary clinics in Surabaya showed that 9.41% of *Escherichia coli* isolates were ESBL producers [14]. These findings suggest that dogs may act as carriers of ESBL‐producing bacteria in both clinical and community‐related settings. However, information on ESBL‐producing *Escherichia coli* in dogs undergoing interregional transport procedures in Indonesia remains very limited.

This knowledge gap is important because transported dogs may serve as carriers of resistant bacteria and contribute to their dissemination across regions. Therefore, this study aimed to detect selected ESBL genes in *Escherichia coli* isolated from rectal swab samples collected from dogs undergoing interregional transport procedures at Juanda Quarantine Station, Surabaya, Indonesia. The findings are expected to provide baseline data for antimicrobial resistance surveillance in companion animals and to support future One Health–based monitoring and control strategies.

## 2. Materials and Methods

### 2.1. Ethical Approval

Rectal swab samples were collected using a noninvasive procedure from privately owned dogs presented at Juanda Quarantine Station for interregional transport requirements. Informed consent was obtained from the animal owners prior to sample collection. Because the sampling procedure was noninvasive and conducted with owner consent, formal ethical approval was not required for this study. Administrative permission for sample collection was obtained from the Juanda Quarantine Authority.

### 2.2. Study Period and Location

This study was conducted at the Juanda Quarantine Station, East Java, Indonesia. Rectal swab samples were collected from dogs scheduled for interregional transport through Juanda International Airport between March and May 2025, with a total of 113 dogs included in the study.

### 2.3. Animal Group

This study involved privately owned dogs undergoing interregional transport procedures through Juanda Quarantine Station, Surabaya, Indonesia. The animals were presented as part of the routine administrative and health requirements for transport and were not quarantined because of suspected illness. Rectal swab sampling was performed after the owners had provided informed consent. Based on the available records, the dogs had undergone routine health examination prior to transport; however, detailed epidemiological information such as age, sex, breed, origin, and prior antimicrobial exposure was not comprehensively available. Therefore, analysis of differences in ESBL gene detection according to these characteristics could not be performed.

### 2.4. Isolation and Identification of *Escherichia coli*

Rectal swab samples from dogs were placed in centrifuge tubes containing 10 mL of Buffered Peptone Water (BPW) (Oxoid, UK). The samples were transported in a cool box maintained at 4°C to the Veterinary Microbiology and Immunology Laboratory, Faculty of Veterinary Medicine, Universitas Brawijaya. The BPW samples underwent 24‐hour incubation at 37°C before eosin methylene blue (EMB) agar (Merck, Germany) inoculation. Gram staining was performed according to the method described by Paray et al. [15]. The isolates were subsequently identified using biochemical tests, including triple sugar iron agar (Merck, Germany), urease (Oxoid, UK), indole motility (Merck, Germany), Voges–Proskauer (Merck, Germany), citrate (Oxoid, UK), and methyl red (Merck, Germany).

### 2.5. Detection of Selected ESBL Genes in *Escherichia coli*

Confirmed *Escherichia coli* isolates were subjected to polymerase chain reaction (PCR) for the detection of selected ESBL genes (*blaCTX-M*, *blaTEM*, and *blaSHV*). Prior to DNA extraction, the isolates were cultured on nutrient agar (Oxoid, UK) and incubated at 37°C for 24 h. Genomic DNA was extracted using the QIAamp DNA Mini Kit (Qiagen, Germany) according to the manufacturer’s instructions. Briefly, bacterial cells were lysed, transferred to a spin column, and washed by centrifugation at 6000 × g and 20,000 × g, and the DNA was eluted in 200 µL Buffer AE. PCR amplification was performed using GoTaq Green Master Mix (Promega, USA) in a total reaction volume of 25 µL, consisting of 12.5 µL GoTaq Green Master Mix (2x), 0.5–2.5 µL of each primer (10 µM), 2 µL DNA template, and nuclease‐free water to the final volume. Multiplex PCR was used for the detection of *blaCTX-M* and *blaTEM*, while *blaSHV* was detected using singleplex PCR. The thermocycling conditions for multiplex PCR consisted of an initial denaturation at 94°C for 2 min, followed by 30 cycles of denaturation at 94°C for 1 min, annealing at 52°C for 30 s, extension at 72°C for 45 s, and a final extension at 72°C for 5 min. For singleplex PCR, amplification was performed with an initial denaturation at 95°C for 5 min, followed by 35 cycles of denaturation at 95°C for 1 min, annealing at 56°C for 1.5 min, extension at 72°C for 1 min, and a final extension at 72°C for 10 min. PCR products were analyzed by agarose gel electrophoresis using ultrapure agarose (Invitrogen, USA). Primer details for each gene are listed in Table 1.

**TABLE 1:** Primers used for ESBL gene detection.

ESBL gene	Primary sequence	Expected size (bp)	References
TEM‐F	5′‐ATA‐AAA‐TTC‐TTG‐AAG‐ACG‐AAA‐3′	1086	[16]
TEM‐R	5′‐GAC‐AGT‐TAC‐CAA‐TGC‐TTA‐ATC‐3′		
SHV‐F	5′‐GGG TTA TTC TTA TTT GTC GC‐3′	928	[17]
SHV‐R	5′‐TTA GCG TTG CCA GTG CTC‐3′		
CTX‐M‐F	5′‐CGC‐TTT‐GCG‐ATG‐TGC‐AG‐3′	550	[18]
CTX‐M‐R	5′‐ACC‐GCG‐ATA‐TCG‐TTG‐GT‐3′		

Abbreviations: bp, base pair; ESBL, extended‐spectrum beta lactamase.

## 3. Results and Discussion

**FIGURE 1:**
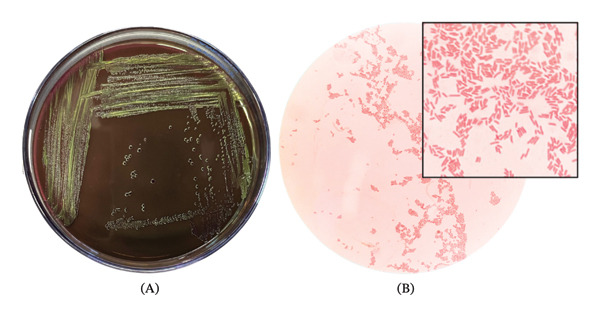
*Escherichia coli* with metallic green color on eosin methylene blue agar (A); Gram staining (1000 × magnification), Gram‐negative, short rod‐shaped bacteria (B).

**FIGURE 2:**
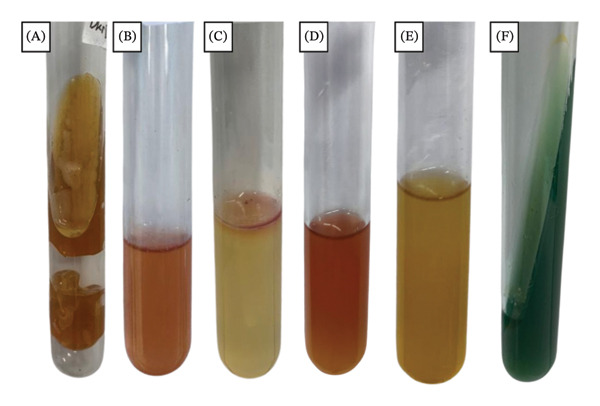
Representative biochemical test results of *Escherichia coli* isolates: (A) triple sugar iron agar showing an acid slant and acid butt (A/A), gas production, and no H_2_S production; (B) negative urease test; (C) sulfide–indole–motility test showing no H_2_S production, positive indole production, and nonmotility; (D) positive methyl red test; (E) negative Voges–Proskauer test; and (F) negative citrate utilization test.

**FIGURE 3:**
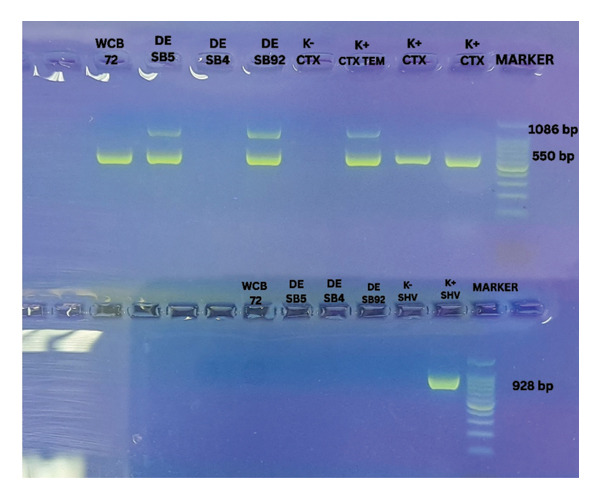
ESBL‐positive confirmed by PCR assay. Multiplex PCR was used for the detection of *blaCTX-M* (550 bp) and *blaTEM* (1086 bp), while *blaSHV* (928 bp) was detected using singleplex PCR. Lane designations include clinical isolates (WCB 72, DE SB5, DE SB4, and DE SB92); negative control (K−); positive control (K+), marker size are indicated on the right. K+ CTX‐M: *Escherichia coli* ESBL (EQASIA 2021/E 21.4), K+ TEM: *Escherichia coli* ATCC 35218, K + SHV: *Klebsiella pneumoniae* ATCC 700603, K−: *Escherichia coli* ATCC 25922.

Based on the sample analysis, 85 of 113 samples (75.22%) tested were positive for *Escherichia coli*. Colonies exhibited a characteristic metallic green sheen on EMB agar (Figure 1A) and were considered presumptive *Escherichia coli* isolates [19]. The morphology observed by Gram staining showed Gram‐negative, short rod‐shaped bacteria (Figure 1B) [20]. Biochemical identification was subsequently performed as presented in Figure 2. PCR‐based detection of ESBL‐associated genes confirmed that 3 of 85 *Escherichia coli* isolates (3.53%) were positive for ESBL genes (Figure 3). Of these positive isolates, 2/3 (66.67%) harbored a combination of *blaCTX-M* and *blaTEM*, while 1/3 (33.33%) carried *blaCTX-M* alone (Table 2). No *blaSHV* gene was detected.

**TABLE 2:** ESBL gene profiles detected by PCR.

ESBL genotype	Number of isolates	
	*n*	%
TEM	—	—
CTX‐M	1	33.33
TEM + CTX‐M	2	66.67
SHV	—	—
Total	3	100

The predominance of *blaCTX-M*, either alone or in combination with *blaTEM*, is consistent with previous reports indicating that CTX‐M‐type ESBLs are among the most widespread ESBL determinants in *Escherichia coli* from humans and animals [6, 13]. The co‐occurrence of *blaCTX-M* and *blaTEM* observed in this study may reflect the role of mobile genetic elements, particularly plasmids, in the accumulation and dissemination of multiple resistance determinants within the same isolate [8]. According to the findings by Agustin et al. [21], *blaTEM* is also widespread in wildlife and has been found in bats, indicating the potential for interspecies dissemination of resistance. This ESBL gene can spread to other bacteria through conjugation, transformation, or transduction [22]. In contrast, the absence of *blaSHV* in the present study may be related to its lower occurrence in canine *Escherichia coli* isolates or to regional differences in circulating ESBL genotypes. Although *blaSHV* has been reported in *Escherichia coli* from broiler farms, beef cattle, and occasionally dogs, it appears to be less frequent than CTX‐M‐type ESBLs in many studies [23–25]. Furthermore, Dameanti et al. [26] reported that *blaSHV* is more commonly found in *Klebsiella pneumoniae* isolates.

The prevalence observed in this study (3.53%) was lower than that reported by Kristianingtyas et al. [14], who found 9.41% ESBL‐producing *Escherichia coli* among rectal swab isolates from dogs in veterinary clinics in Surabaya. It was also lower than the average prevalence reported in the Indonesian meta‐analysis by Kadariswantiningsih et al. [27], which documented substantial heterogeneity in ESBL‐producing bacteria across studies. However, this lower prevalence should not be interpreted as negligible. Even a relatively low prevalence remains epidemiologically important because ESBL genes are transferable antimicrobial resistance determinants that may be maintained by apparently healthy carrier animals [28]. In the present study, the positive dogs were sampled in the context of interregional transport procedures, which increases the relevance of the finding because transported animals may contribute to the movement of resistant bacteria or resistance genes across geographic areas.

The significance of the 3.53% positivity rate should therefore be interpreted in terms of dissemination risk rather than frequency alone. A small number of positive animals may still be important from a public health perspective when they belong to a host population that lives in close contact with humans and can move between regions. Dogs are companion animals that commonly share household environments with humans and may facilitate microbial exchange through direct contact or indirect environmental contamination [12]. Previous studies have reported identical phylogenetic groups, antimicrobial susceptibility patterns, and ESBL gene profiles in *Escherichia coli* isolates obtained from dog–owner pairs, suggesting the possibility of interspecies transmission [29]. Thus, the present result is significant not because the prevalence was high, but because the detected bacteria were found in a mobile companion‐animal population with the potential to disseminate antimicrobial resistance determinants beyond a single location.

These findings are also relevant within a One Health framework, which recognizes the interconnection among human, animal, and environmental health in the emergence and spread of antimicrobial resistance [10, 28]. Companion animals occupy an important position in this framework because they interact closely with humans while also contributing to environmental contamination through fecal shedding [30]. Dog feces may contain ESBL‐producing *Escherichia coli* and contaminate households, public spaces, and the surrounding environment, creating opportunities for resistant bacteria to circulate between animals, humans, and environmental reservoirs [31]. In addition, dog feces may also contain other zoonotic pathogens, highlighting the broader public health importance of proper hygiene and waste management in companion animal settings [32]. Maintaining and improving hygiene plays a crucial role in preventing the further spread of antibiotic resistance genes [33]. Therefore, the detection of ESBL‐associated genes in dogs at a quarantine‐related transport setting should not be viewed solely as a veterinary issue, but also as a relevant One Health concern.

Previous studies have shown that ESBL‐associated genes may be detected in both healthy and diarrheic dogs, with *blaCTX-M* and *blaTEM* among the most frequently detected genes [9, 34]. This supports the possibility that apparently healthy dogs may act as carriers of ESBL‐producing bacteria without obvious clinical signs. In the present study, the sampled animals were undergoing transport‐related procedures rather than being sampled because of suspected illness, which reinforces the concern that clinically normal companion animals may still contribute to the silent dissemination of ESBL‐producing *Escherichia coli*.

The broader Indonesian context further supports the relevance of the present findings. Several studies from Indonesia have reported ESBL‐associated genes in animal, environmental, and mixed‐source isolates, including *blaTEM*, *blaCTX-M*, *blaSHV*, and *blaOXA* [27, 35, 36]. These reports suggest that antimicrobial resistance genes are already circulating across multiple interfaces in Indonesia. Accordingly, the detection of ESBL genes in dogs at Juanda Quarantine Station should be considered part of a larger pattern of antimicrobial resistance circulation involving animals, humans, and the environment.

Taken together, the present findings indicate that dogs undergoing interregional transport procedures may serve as carriers of ESBL‐producing *Escherichia coli*. This underscores the importance of prudent antibiotic use in companion animals and strengthened antimicrobial stewardship in veterinary practice [10]. From a One Health perspective, monitoring ESBL‐producing bacteria in companion animals is important not only for animal health but also for reducing the risk of dissemination to humans and the environment. The present study therefore provides baseline evidence supporting the inclusion of companion animals, particularly transported pets, in antimicrobial resistance surveillance efforts in Indonesia.

## 4. Conclusion

This study demonstrated the presence of selected ESBL genes in *Escherichia coli* isolated from dogs undergoing interregional transport procedures at Juanda Quarantine Station, Surabaya, Indonesia. Of 85 *Escherichia coli* isolates, 3 (3.53%) carried ESBL‐associated genes, with *blaCTX-M* detected alone or in combination with *blaTEM*. Although the prevalence was relatively low, the finding remains important because transported dogs may serve as carriers of antimicrobial resistance determinants and may contribute to their dissemination across regions. These results support the inclusion of companion animals in antimicrobial resistance surveillance and reinforce the importance of a One Health approach in efforts to mitigate the spread of antimicrobial resistance among humans, animals, and the environment.

## 5. Limitation of the Study

This study has several limitations. First, the sample size was limited to dogs available during the study period at a single quarantine station, which may restrict the generalizability of the findings. Second, the study focused on the detection of selected ESBL genes and did not include sequencing‐based confirmation, plasmid typing, or minimum inhibitory concentration (MIC) testing. Third, detailed epidemiological information, including age, sex, breed, origin, and prior antimicrobial exposure, was not comprehensively available for all sampled dogs. Finally, this study did not include comparative sampling from owners or environmental sources; therefore, direct transmission pathways and the relatedness of bacterial isolates across different compartments could not be determined.

## Author Contributions

This section mentions the assignment of each author, including tasks such as conceptualization, Mustofa Helmi Effendi, methodology, Wiwiek Tyasningsih, software, Dadik Rahardjo and Fidi Nur Aini Eka Puji Dameanti, validation, Boedi Setiawan, formal analysis, Benny Aprisa Surya Perdana, investigation, Benny Aprisa Surya Perdana, resources, Benny Aprisa Surya Perdana, data curation, Wiwik Misaco Yuniarti, writing–original draft, Benny Aprisa Surya Perdana, writing–review and editing, Fidi Nur Aini Eka Puji Dameanti, visualization, Boedi Setiawan, supervision, Mustofa Helmi Effendi and Boedi Setiawan, project administration, Dadik Rahardjo, and funding acquisition, Benny Aprisa Surya Perdana.

## Funding

This research is funded by the Indonesian Endowment Fund for Education (LPDP) on behalf of the Indonesian Ministry of Higher Education, Science, and Technology and managed under the EQUITY Program (Contract Nos. 4300/B3/DT.03.08/2025 and 297/UN3/HK.07.00/2025) and International Research Network Scheme (No. 5452/B/UN3).

## Conflicts of Interest

The authors declare no conflicts of interest.

## Data Availability

The datasets used and analyzed in this study are available from the corresponding author upon reasonable request.
